# Perceptual holistic color combination analysis of *Papilionidae* butterflies as aesthetic objects

**DOI:** 10.1371/journal.pone.0240356

**Published:** 2020-10-28

**Authors:** Erina Kakehashi, Keiichi Muramatsu, Haruo Hibino

**Affiliations:** 1 Global Research Institute, Keio University, Tokyo, Japan; 2 Global Education Center, Waseda University, Tokyo, Japan; 3 Graduate School of Engineering, Chiba University, Chiba, Japan; Universita degli Studi di Perugia, ITALY

## Abstract

In this study, we clarified the holistic color combination rules of human-preferred *Papilionidae* butterflies by examining the hue, lightness, and chroma. A set of 118 *Papilionidae* butterfly images used in our previous study was analyzed. These images were classified via hierarchical density-based spatial clustering based on perceptual similarities of colors that were obtained from a subjective image classification experiment. The color combinations of the clustered images were determined based on representative colors that were analyzed by a Gaussian mixture model with minimum message length and the color combination types defined in our previous study. Consequently, we obtained the following holistic color combination rules for *Papilionidae*: 1) contrasting lightness, similar chroma, and similar hue, 2) contrasting lightness, contrasting chroma, and similar hue, 3) similar lightness, similar chroma, and complementary hue, and 4) similar lightness, similar chroma, and similar hue. These rules suggest that minority color harmony theories are valid under particular conditions.

## Introduction

Color harmony studies contribute not only to developing color design but also to clarifying the mechanisms of human aesthetic responses. Conventional color harmony theories developed in the past were not always consistent with psychological results [1–4]. Thus, to achieve consistency between conventional color harmony theories and psychological results, the concept of computational aesthetics has had to be applied to color harmony studies, e.g., clarifying the color combination structures of paintings and butterflies as aesthetic objects [5, 6]. In a previous study, the common color combination rules in paintings were not clarified [5]. In contrast, our previous work [6] focused on the beautiful colors in nature that have been applied to color design [7–11], and the artistic quality of *Papilionidae* butterflies [12–17]. Therein, the color combination rules of 118 human-preferred *Papilionidae* butterfly images were clarified [6], i.e., contrasting lightness, similar chroma, and similar hue, which agreed with a part of the psychological color harmony principles [1–4]. Nevertheless, similar lightness, contrasting chroma, and complementary hue have also been obtained as a minor proportion of results that agree with a part of the conventional color harmony principles [1, 18]. These results suggest that the minority color combinations that do not appear in the psychological results may harmonize limitedly. The color combinations were analyzed according to the color appearance attributes (i.e., lightness, chroma, and hue) independently and were considerably simplified in our previous work. To obtain more detailed color combination rules, the color combinations should be analyzed according to the integrated color appearance attributes. Moreover, in our previous work, the color combinations of classified images based on color similarities were analyzed [6]. Our previous work used a computational image classification method, which did not reflect the human visual perception accurately. To obtain more perceptual results, the color similarities should be measured by a human.

Accordingly, in this study, we aimed to clarify the holistic color combination rules of human-preferred *Papilionidae* butterflies according to the integrated lightness, chroma, and hue. Furthermore, to implement a more perceptual analysis, we applied color similarities based on human visual perception to a color combination analysis method.

## Method

In this study, the method developed in our previous study was improved while preserving its framework [6]. Initially, a subjective image classification experiment was conducted to obtain the perceptual color similarities of images. Subsequently, these similarities were used as Hierarchical Density-Based Spatial Clustering of Applications with Noise (HDBSCAN) variables to classify the images. Then, the color distributions of the image clusters were segmented using the Gaussian mixture model (GMM) to extract representative colors of each cluster. Finally, the positional relations of the representative colors of a cluster on a color space were compared with the color combination types defined in our previous study to determine the color combination characteristics of the clusters.

This study was approved by the ethics committee of Chiba University.

### Image classification experiment

To determine the perceptual similarity between each pair of images, we conducted a subjective image classification experiment. Experimental methods to measure the perceptual similarities of images include the following: (a) table scaling experiment, in which observers arrange the stimuli on the table according to their perceptual similarities; (b) computer scaling experiment, where a reference image is compared with some images, and the most similar one to the reference image is selected [19]; and (c) ViSiProG test, wherein some stimuli are presented simultaneously, and observers select and place the similar stimuli in a separate box to form the clusters [20]. To classify images of similar colors, as required in this study, a modified version of the ViSiProG test was designed.

**Fig 1:**
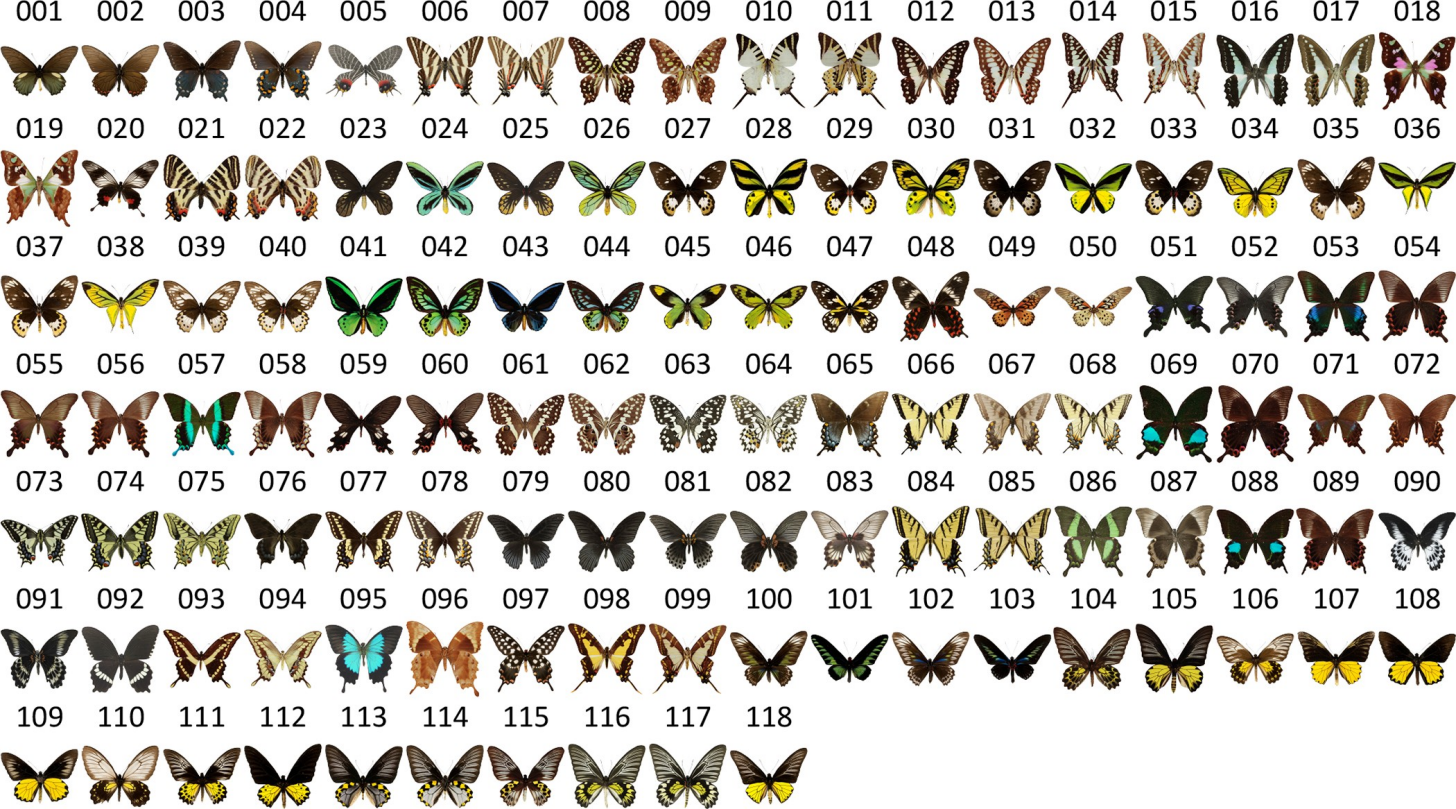
118 images of butterflies of the *Papilionidae* family. The corresponding code numbers are included with each image.

We selected 118 *Papilionidae* butterfly images, which were used in our previous work as human-preferred *Papilionidae* butterflies (Fig 1) [6], as the stimuli. These images were centered on a white square-background (R, G, B = 1.0, 1.0, 1.0) covering an area of 12 cm^2^.

**Fig 2:**
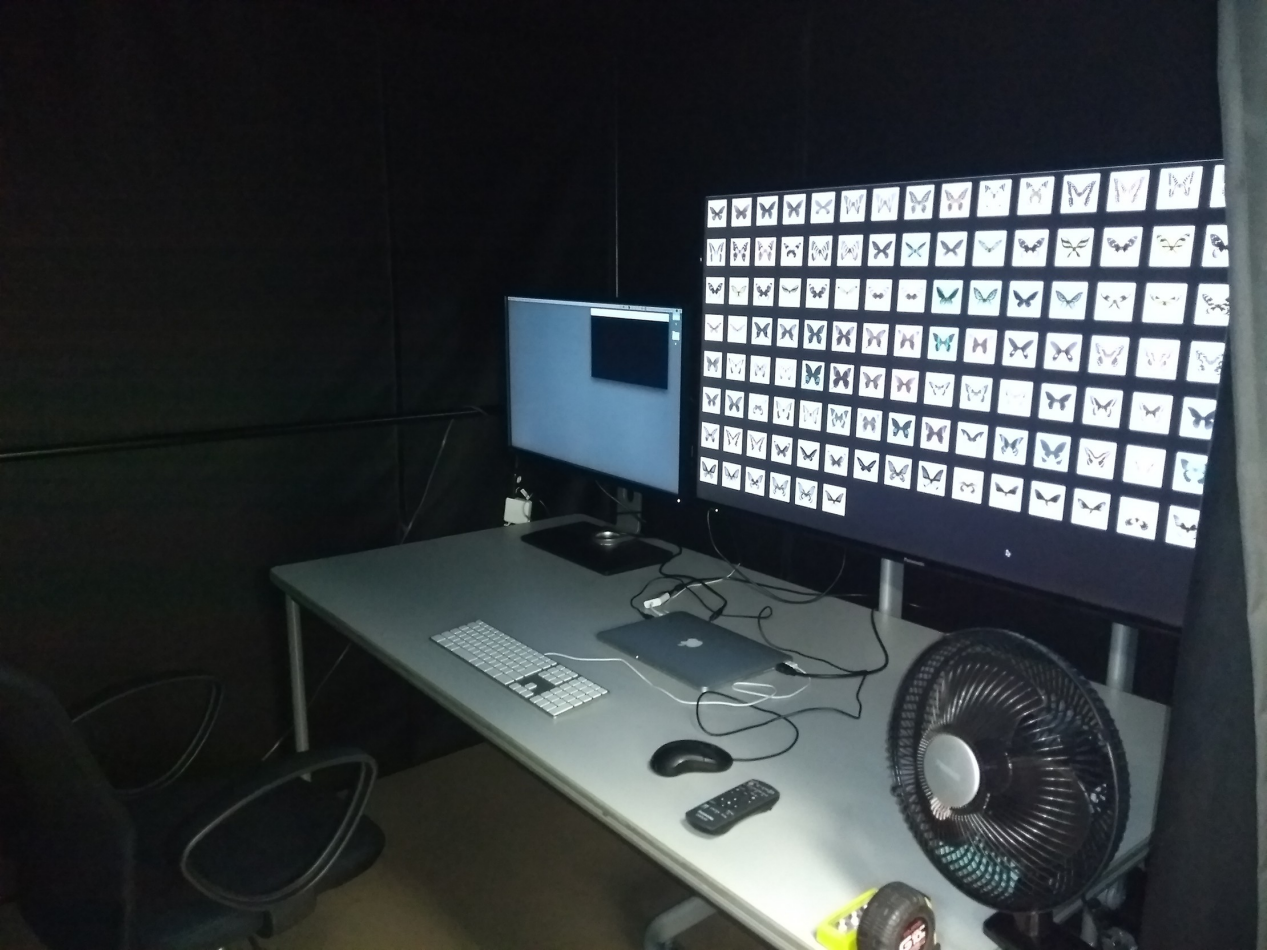
Experimental environment.

**Fig 3:**
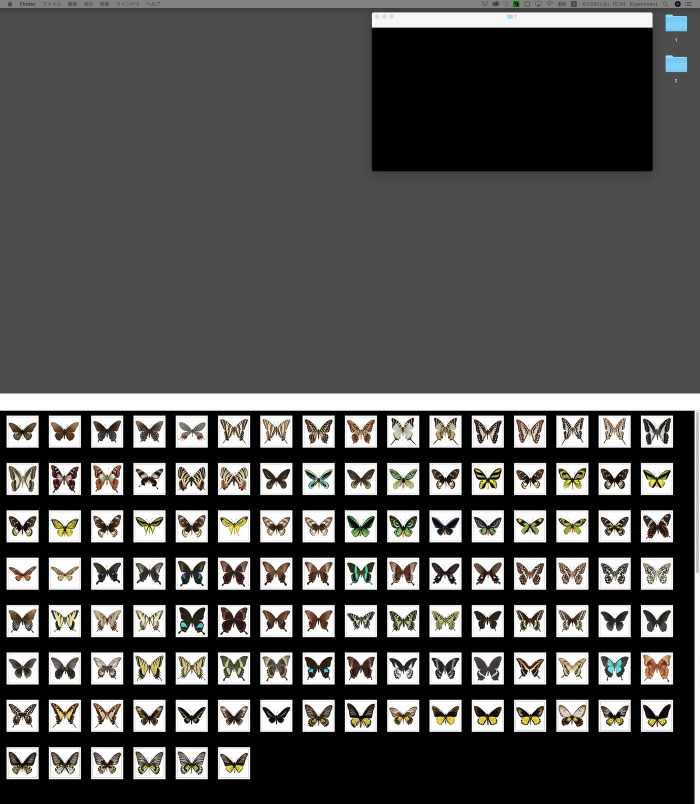
Experiment window. (a) Folder area on the left monitor. (b) Thumbnail images displayed on the right monitor.

As the experimental environment, two monitors (50 in and 32 in) connected to a MacBook Pro (Retina, 13-inch, Early 2015) (Apple, Inc., Cupertino, CA, USA) were placed in a darkened room (Fig 2). Additionally, a 50 in monitor (TH-50LFE7J, Panasonic, Corp., Osaka, Japan) was positioned to the right of a 32 in monitor (BL3201PT, BenQ, Inc., Taipei, Taiwan). An i1 Display Pro (X-Rite, Inc., Grand Rapids, MI, USA) was used to correct the colors of both monitors. On the 50 in monitor, thumbnail images of all stimuli were presented simultaneously against a black background (R, G, B = 0, 0, 0). The arrangement was in random order and the size was 5 cm^2^ (Fig 3(A)). Those images were enlarged to 12 cm^2^ upon double-clicking. On the 32 in monitor, the enlarged images and two folders were presented, and one folder was opened (Fig 3(B)). The folders had a black background (R, G, B = 0, 0, 0) and displayed no extra information (e.g., toolbar and sidebar). The left (32 in) monitor’s background was “Solid Gray Pro Ultra Dark” (R, G, B = 0.188, 0.188, 0.188) in Mac. The viewing distance between the monitor and the observer was 75 cm. Thirty-one observers with normal color vision from Chiba University participated in the experiment (16 males and 15 females, aged from their late teens to 30s).

In the experiment, each observer took Ishihara’s test for color deficiency and was taught how to operate the experimental window. They were instructed to classify the stimuli based on only color, independent of shape and pattern. During the experiment, each observer dragged similarly colored images from the right monitor into the same folder on the left monitor. They could not include the same stimulus in the other folders, but could add new folders as necessary. This trial was repeated until all 118 stimuli were classified. The observers were briefed on the experiment and its safety, and provided written consent before beginning the experiment. For minor observers, consent from parents or guardians was not required by the ethics committee.

### Image classification

To classify the 118 *Papilionidae* images, we calculated the similarities of the images based on the experimental results. First, to obtain the similarities, the frequencies at which a pair of images was classified into the same folders in the experiment were divided by the number of observers. The distance matrix was defined by the similarity of each pair of images subtracted from 1. The matrix consisted of 117 rows and columns, and each element of this matrix denotes the distance of each pair of images. Table 1 shows a part of the distance matrix as an example.

**Table 1:** A part of the distance matrix.

	001	002	003	004	005	006	007	008	009
002	0.806								
003	0.548	0.871							
004	0.742	0.516	0.677						
005	0.903	0.839	0.935	0.839					
006	0.968	1	1	1	0.968				
007	0.935	1	1	1	0.935	0.258			
008	0.903	0.968	0.903	0.935	0.903	0.677	0.613		
009	0.871	0.839	0.968	0.903	0.935	0.774	0.613	0.806	
010	0.935	1	0.968	1	0.935	0.774	0.742	0.839	0.742

Distance of each pair of images coded from 001 to 010 is shown as a lower triangle matrix.

Using the distance matrix, HDBSCAN was performed with the dbscan package of R (The R Foundation, Vienna, Australia) to classify the images [21]. The minimum size of the clusters was two. HDBSCAN is a hierarchically modified density-based spatial clustering of applications with noise (DBSCAN) [22]. It can automatically determine the clusters while removing noise. Because HDBSCAN improve the hierarchical cluster analysis used in our previous work, it was used in this study.

### Color combination characteristics

In our previous work, the representative colors of the classified images were extracted by a GMM with the mclust package of R (The R Foundation, Vienna, Australia) [23]. The GMM considers the color distributions as mixed multiple Gaussians and presents the probability density function of the color distributions in each Gaussian mixture component by using three variables, namely, mean values (μ), mixing proportions (πk, the estimated populations that configure a Gaussian), and covariance matrix (Σ), which were considered as the values, sizes, and ranges of a representative color, respectively. In this work, because the proportions under 3% were almost invisible [24], they were excluded from the analysis [6]. The number of components in each cluster was determined from the minimum message length criterion (MML) instead of the Bayesian information criterion (BIC), which was used as the reference value in our previous work [6]). The MML values were calculated for 20 components, because further components would complicate the results. In the MML, the shorter the code build for the data, the better the data generation models are [25]. GMM with MML outperformed those with other criterion, including BIC, in estimating the number of components [25]. Furthermore, it was effective in unsupervised color image segmentation with GMM and automatic estimation of the number of components [26]. Therefore, because GMM with MML improves upon the results obtained in our previous work using GMM with BIC, it was used in this study.

To determine the color combination characteristics of each cluster, we used the same color combination types as defined in our previous work (Table 2) [6]. The representative colors were classified into any one of the categories listed in Table 2 based on the mean values in the CIELCh color space (lightness, chroma, and hue scale in CIELAB) [27]. The color combinations of the clusters were determined from the color combination types in Table 2 based on the category combinations of representative colors.

**Table 2:** Defined color categories and color combination types in our previous work.

Range	Category	Similar	Contrast
L*, C*_ab_ < = 33	Low	Low	High
33 < (L*, C*_ab_) < = 66	Middle	Middle	-
66 < L*, C*_ab_	High	High	Low
0 < = *h*_*ab*_ < 30	1	2, 3, 11, 12	7
30 < = *h*_*ab*_ < 60	2	1, 3, 4, 12	8
60 < = *h*_*ab*_ < 90	3	1, 2, 4, 5	9
90 < = *h*_*ab*_ < 120	4	2, 3, 5, 6	10
120 < = *h*_*ab*_ < 150	5	3, 4, 7, 8	11
150 < = *h*_*ab*_ < 180	6	4, 5, 7, 8	12
180 < = *h*_*ab*_ < 210	7	5, 6, 8, 9	1
210 < = *h*_*ab*_ < 240	8	6, 7, 9, 10	2
240 < = *h*_*ab*_ < 270	9	7, 8, 10, 11	3
270 < = *h*_*ab*_ < 300	10	8, 9, 11, 12	4
300 < = *h*_*ab*_ < 330	11	1, 9, 10, 12	5
330 < = *h*_*ab*_ < 360	12	1, 2, 10, 11	6

“Similar” indicates similar color categories, and “Contrast” shows the contrasting color categories.

## Results

**Fig 4:**
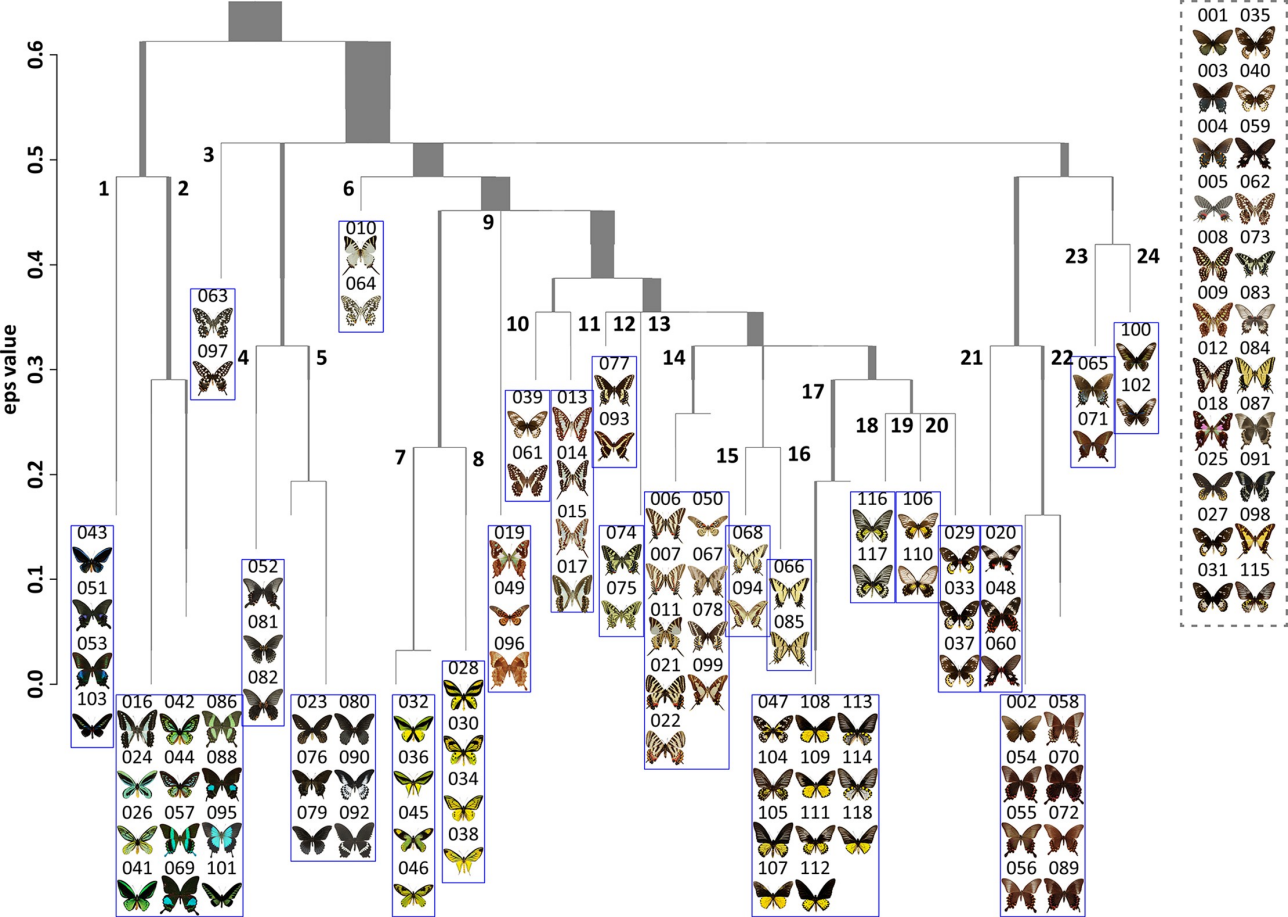
Simplified dendrogram showing 24 clusters of 118 images obtained by HDBSCAN. The eps value shows the value of the epsilon neighborhood parameter. The width of the viatical line shows the number of points (images) in the cluster. Blue rectangles show each cluster. Gray dotted-rectangle shows the images removed as noise.

The selected images were classified into 24 clusters based on their perceptual similarity. Fig 4 depicts the dendrogram of the classified images and their cluster numbers. Table 3 lists the number of components (representative colors) and the best MML values in each cluster. Table 4 shows the μ, πk, and Σ values of each component in the clusters.

**Table 3:** Number of components (“G”) in each cluster (“No.”) selected by GMM.

No.	MML	model	G
1	2729463	VVV	18
2	9626306	VVI	20
3	1237900	VVV	20
4	1687101	VVV	19
5	3472926	VVV	20
6	1391970	VVV	20
7	1939729	VVV	14
8	2309471	VVE	9
9	2203279	VVV	18
10	1388672	VVV	17
11	3116491	VVV	17
12	1123383	VVV	20
13	1371536	VVV	17
14	6779642	VVV	19
15	1203188	VVV	15
16	1276463	VVV	13
17	7602091	VVV	10
18	1508144	VVV	16
19	1328840	VVV	20
20	1918051	VVV	14
21	1929157	VVV	19
22	6122828	VVV	18
23	1426978	VVV	19
24	1142360	VVV	17

The model is parameterized by geometric characteristics determined using the covariance matrix of each component. “VVV” shows an ellipsoidal distribution and varying volume, shape, and orientation. “VVI” shows a diagonal distribution and varying volume and shape. “VVE” shows an ellipsoidal distribution and equal orientation.

**Table 4:** Proportions (“πk”), mean values (“μ”), and covariance matrices (“∑”) for each component (“No”).

**(a)**					
**Cluster 1**					
**No**	**πk**	**μ**	**Σ**		
			L^*^	C^*^_ab_	***h***_**ab**_
1	0.163	2.833	2.79	1.59	22.9
		2.294	1.59	1.59	17.3
		42.51	22.9	17.3	508
2	0.0722	5.972	7.67	6.08	36.5
		6.402	6.08	9.19	21.6
		37.4	36.5	21.6	361
3	0.0625	12.24	22.4	16.3	35.9
		17.04	16.3	24.6	16.8
		40.8	35.9	16.8	105
4	0.1	8.389	10.3	4.21	17.3
		4.805	4.21	4.41	10.1
		79.04	17.3	10.1	1310
5	0.0413	17.71	51.3	6.14	63.3
		5.704	6.14	4.65	32
		71.43	63.3	32	730
6	0.0687	20.08	44.9	23.4	34
		14.37	23.4	31.2	-42.5
		82.98	34	-42.5	726
7	0.083	1.552	0.672	0.218	-3.34
		0.9651	0.218	0.362	-7.94
		91.81	-3.34	-7.94	656
8	0.088	15.67	19.6	11	4.48
		10.92	11	15	15.7
		119.6	4.48	15.7	138
9	0.0525	27.34	38.6	6.22	-17.8
		16.87	6.22	27.6	35.3
		120.3	-17.8	35.3	98
10	0.0474	12.94	40.4	15.3	-108
		7.893	15.3	21.5	-28
		265.6	-108	-28	2270
11	0.0766	36.52	193	108	-15.5
		32.55	108	141	82.6
		271.9	-15.5	82.6	174
12	0.031	47.16	180	146	69.7
		35.23	146	196	108
		130	69.7	108	180
**(b)**					
**Cluster 2**					
**No**	**πk**	**μ**	**Σ**		
			L^*^	C^*^_ab_	***h***_**ab**_
1	0.117	4.556	2.91	0	0
		5.398	0	5.77	0
		34.36	0	0	255
2	0.128	23.88	49.3	0	0
		10.51	0	44.3	0
		78.69	0	0	687
3	0.07	1.882	1.82	0	0
		1.176	0	0.733	0
		73.98	0	0	2760
4	0.0822	8.86	6.58	0	0
		6.456	0	2.74	0
		77.18	0	0	548
5	0.122	11.79	11.2	0	0
		12.47	0	17.3	0
		68.06	0	0	1190
6	0.0306	20.72	18.2	0	0
		24.44	0	30.5	0
		131.9	0	0	149
7	0.0328	33.75	63.8	0	0
		23.75	0	22.8	0
		110.7	0	0	32.7
8	0.0584	45.85	193	0	0
		26.18	0	145	0
		133.7	0	0	2860
9	0.0413	43.34	93.5	0	0
		40.8	0	72	0
		131.1	0	0	114
10	0.0303	80.14	11.6	0	0
		42.71	0	38.1	0
		130.7	0	0	33.9
11	0.0701	68.23	53.3	0	0
		63.41	0	114	0
		129	0	0	233
12	0.0609	73.45	40.3	0	0
		35.23	0	34.2	0
		170.8	0	0	764
**(c)**					
**Cluster 3**					
**No**	**πk**	**μ**	**Σ**		
			L^*^	C^*^_ab_	***h***_**ab**_
1	0.0883	19.16	16.5	0.298	-2.38
		13.63	0.298	0.833	0.275
		65.88	-2.38	0.275	9.27
2	0.0663	17.72	8.33	-0.504	1.63
		9.96	-0.504	0.139	0.142
		82.51	1.63	0.142	0.858
3	0.0417	23.85	130	40.3	-60.1
		10.09	40.3	16.4	-18
		80.74	-60.1	-18	175
4	0.0784	19.89	37.8	12	13.9
		13.92	12	21.1	5
		65.92	13.9	5	51.2
5	0.0701	20.78	41.4	0.82	23
		10.09	0.82	2.53	8.02
		81.81	23	8.02	40.8
6	0.0704	7.679	7.57	5.08	13.6
		7.428	5.08	3.9	11.2
		68.03	13.6	11.2	66.6
7	0.0544	11.4	8.11	4.06	15.2
		12.11	4.06	2.89	7.52
		66.4	15.2	7.52	60
8	0.123	12.62	26.9	9.85	12.2
		8.134	9.85	7.04	7.54
		72.55	12.2	7.54	172
9	0.0378	15.29	16.5	-0.227	1.92
		6.538	-0.227	0.288	0.425
		90.64	1.92	0.425	10.5
10	0.0743	29.8	108	9.35	27.8
		9.609	9.35	5.41	7.51
		93.19	27.8	7.51	55
11	0.0327	48.05	234	30.1	41.6
		14.38	30.1	19.6	5.12
		101.8	41.6	5.12	17.3
12	0.0315	66.7	228	-12.2	62.3
		14.71	-12.2	29	4.31
		77.38	62.3	4.31	65
13	0.0684	92.59	24.9	-5.51	56.3
		5.221	-5.51	6.85	-5.93
		94.95	56.3	-5.93	271
14	0.0418	95.47	2.39	-0.997	0.696
		7.412	-0.997	3.41	-0.618
		117.1	0.696	-0.618	3.67
**(d)**					
**Cluster 4**					
**No**	**πk**	**μ**	**Σ**		
			L^*^	C^*^_ab_	***h***_**ab**_
1	0.0358	6.286	5.79	1.72	-0.357
		3.2	1.72	0.672	2.41
		60.73	-0.357	2.41	111
2	0.0438	7.241	1.16	0.327	0.801
		3.571	0.327	0.0931	0.218
		64.31	0.801	0.218	0.646
3	0.102	16.38	32.6	2.06	14.1
		5.917	2.06	1.62	7.68
		86.42	14.1	7.68	64.4
4	0.0938	12.73	8.18	-0.215	1.82
		3.802	-0.215	0.047	0.572
		65.54	1.82	0.572	9.69
5	0.041	30.83	30.6	-1.27	3.22
		9.134	-1.27	0.171	0.158
		83.99	3.22	0.158	1.07
6	0.0556	10.95	37.4	4.57	73.9
		3.471	4.57	1.74	19.4
		73.47	73.9	19.4	972
7	0.0501	19.82	54.9	-1.86	19.2
		3.81	-1.86	0.306	3.01
		64.22	19.2	3.01	91.7
8	0.0998	25.42	56.1	-3.77	31.9
		8.309	-3.77	4.73	-1
		86.85	31.9	-1	72.2
9	0.0405	22.1	42.6	-1.5	4.83
		6.511	-1.5	0.307	0.744
		64.23	4.83	0.744	33.9
10	0.0871	19.51	19.3	-0.501	2.81
		6.451	-0.501	0.181	0.351
		90.32	2.81	0.351	1.49
11	0.0806	43.46	141	2.24	54.1
		10.05	2.24	2.18	7.74
		89.34	54.1	7.74	54
12	0.0774	50.92	202	-6.34	7.43
		9.017	-6.34	0.737	0.586
		101.1	7.43	0.586	4.02
13	0.0594	55.72	370	-4.77	240
		5.594	-4.77	2.98	-0.458
		95.27	240	-0.458	340
14	0.0421	44.25	141	-2.62	5.94
		5.723	-2.62	0.185	0.278
		91.78	5.94	0.278	1.39
**(e)**					
**Cluster 5**					
**No**	**πk**	**μ**	**Σ**		
			L^*^	C^*^_ab_	***h***_**ab**_
1	0.0537	23.24	6.59	0.0963	3.08
		3.173	0.0963	0.268	2.68
		68.12	3.08	2.68	121
2	0.0714	22.98	98	8.45	136
		3.984	8.45	3.31	39.9
		60.53	136	39.9	790
3	0.0439	12.57	7.09	-0.168	1.89
		3.806	-0.168	0.0452	0.571
		65.52	1.89	0.571	9.71
4	0.122	11.76	10.4	1.51	0.725
		6.158	1.51	3.37	4.93
		75.65	0.725	4.93	187
5	0.0567	14.29	11.1	4.97	11.9
		10.73	4.97	6.18	10.5
		73.18	11.9	10.5	69
6	0.0438	28.24	97.6	44.4	16.1
		15.52	44.4	30.4	-2.9
		80.1	16.1	-2.9	107
7	0.0963	23.62	16.8	2.39	-42.4
		3.117	2.39	1.68	-7.58
		87	-42.4	-7.58	376
8	0.0647	7.503	8.42	1.62	2.57
		3.156	1.62	2.22	-13.7
		88.21	2.57	-13.7	734
9	0.0786	13.74	6.02	0.517	-4.23
		8.555	0.517	0.545	-0.906
		68.32	-4.23	-0.906	35.9
10	0.0306	29.47	160	0.0283	59.9
		4.049	0.0283	0.572	4.84
		118.2	59.9	4.84	98.8
11	0.0383	19.13	25.7	-0.915	2.99
		6.567	-0.915	0.209	0.32
		90.5	2.99	0.32	1.4
12	0.0606	11.83	17.2	0.000935	-0.00038
		0.001238	0.000935	5.46E-08	-2.6E-08
		117.8	-0.00038	-2.6E-08	2.53E-08
13	0.0736	25.68	73.9	-2.08	67
		5.454	-2.08	4.24	-15.6
		90.19	67	-15.6	381
14	0.0583	49.01	692	-1.83	-743
		3.362	-1.83	2.78	16.1
		243.5	-743	16.1	2460
**(f)**					
**Cluster 6**					
**No**	**πk**	**μ**	**Σ**		
			L^*^	C^*^_ab_	***h***_**ab**_
1	0.0446	3.765	3.48	2.39	18
		3.59	2.39	2.82	13.5
		35.49	18	13.5	282
2	0.0406	3.245	0.532	0.31	-5.58
		2.184	0.31	0.629	-6.56
		56.66	-5.58	-6.56	880
3	0.0309	11.1	22.7	8.3	5.55
		8.432	8.3	9.64	-9.2
		65.11	5.55	-9.2	382
4	0.0458	23.35	31.4	0.659	11.7
		11.89	0.659	1.33	3.26
		89.58	11.7	3.26	14.1
5	0.113	32.78	135	11.1	51.2
		14.82	11.1	11.9	6.09
		88.96	51.2	6.09	56
6	0.0496	33.19	34.2	2.54	15.4
		14.98	2.54	2.49	5.62
		83.87	15.4	5.62	18
7	0.0451	42.72	369	64.9	361
		25.6	64.9	52.5	67
		68.86	361	67	398
8	0.0563	50.96	353	1.62	299
		13.15	1.62	17.5	1.53
		82.68	299	1.53	413
9	0.0544	95.63	0.202	-0.0647	0.0188
		3.766	-0.0647	1.68	-0.0725
		116.8	0.0188	-0.0725	0.549
10	0.0956	80.71	2	-0.346	0.635
		7.569	-0.346	1.08	-0.814
		117.6	0.635	-0.814	15
11	0.0732	95.56	7.6	-2.95	4.24
		5.571	-2.95	6.57	-7.05
		115.5	4.24	-7.05	64.5
12	0.0927	77.56	138	-18.4	30.3
		15.66	-18.4	23.8	-4.13
		104	30.3	-4.13	19.9
13	0.0355	72.58	6.65	-14.9	9.17
		27.68	-14.9	60.2	-22
		106.1	9.17	-22	16.8
14	0.0355	77.39	11.1	-2.2	19.5
		9.278	-2.2	5.78	-13.2
		117.2	19.5	-13.2	99.7
15	0.108	79.36	5.92	-2.47	1.05
		11.23	-2.47	6.82	-4.9
		113.1	1.05	-4.9	17.6
**(g)**					
**Cluster 7**					
**No**	**πk**	**μ**	**Σ**		
			L^*^	C^*^_ab_	***h***_**ab**_
1	0.064	1.019	0.681	0.925	11.7
		1.911	0.925	1.6	17.5
		24.88	11.7	17.5	297
2	0.0756	8.818	9.93	8.36	24.2
		9.87	8.36	12.4	12
		56.7	24.2	12	141
3	0.0858	4.002	2.68	1.86	15
		4.909	1.86	2.99	0.286
		51.97	15	0.286	270
4	0.0589	12.5	32.3	21.1	13.4
		8.931	21.1	20.1	26.2
		88.47	13.4	26.2	1040
5	0.0924	2.289	1.45	0.948	-6.52
		2.137	0.948	1.14	-11.9
		81.35	-6.52	-11.9	1320
6	0.0415	19.67	40	16.7	28.4
		15.1	16.7	30.8	10.6
		65.81	28.4	10.6	67.7
7	0.0939	50.76	267	249	-38.9
		47.59	249	263	-41
		118	-38.9	-41	28.6
8	0.0877	50.11	299	231	3.81
		40.56	231	237	-28.9
		105.2	3.81	-28.9	139
9	0.0615	91.81	1.79	1.71	1.96
		91.32	1.71	1.64	1.88
		97.34	1.96	1.88	2.18
10	0.0352	81.35	55.2	49.2	17.6
		81.71	49.2	44.2	15.2
		93.77	17.6	15.2	17
11	0.138	68.6	22	19	-5.98
		73.13	19	21.8	4.81
		109.9	-5.98	4.81	23.4
12	0.127	66.39	63.3	54.5	-27.8
		66.83	54.5	54.7	-16.3
		105.7	-27.8	-16.3	50.9
**(h)**					
**Cluster 8**					
**No**	**πk**	**μ**	**Σ**		
			L^*^	C^*^_ab_	***h***_**ab**_
1	0.0805	1.088	0.89	0.858	18.9
		1.486	0.858	1.19	24.9
		33.69	18.9	24.9	755
2	0.162	7.64	19.4	13	6.42
		7.53	13	16.6	8.8
		58.2	6.42	8.8	292
3	0.0656	1.763	1.47	1.09	9.61
		1.453	1.09	1.47	12.7
		100.5	9.61	12.7	386
4	0.0337	0.5271	0.202	0.125	0.357
		0.4641	0.125	0.182	0.473
		113	0.357	0.473	14.6
5	0.159	39.15	350	267	-6.96
		29.06	267	289	-2.21
		95.81	-6.96	-2.21	425
6	0.218	72.75	82.3	64.5	-3.14
		70.89	64.5	67.4	-2.46
		101.1	-3.14	-2.46	42
7	0.188	81.06	43.4	38.5	-1.91
		81.45	38.5	34.6	-1.51
		95.41	-1.91	-1.51	18.1
8	0.0714	91.01	1.07	0.956	-0.0187
		90.59	0.956	0.854	0.000303
		96.46	-0.0187	0.000303	1.59
**(i)**					
**Cluster 9**					
**No**	**πk**	**μ**	**Σ**		
			L^*^	C^*^_ab_	***h***_**ab**_
1	0.0345	17.52	17.2	19.2	13.5
		30.87	19.2	30	13.9
		42.34	13.5	13.9	16.6
2	0.0355	10.96	18.1	13.3	32.4
		16.45	13.3	26.9	12.3
		37.37	32.4	12.3	93.5
3	0.103	30.67	55.3	17.7	35.1
		38.47	17.7	18.2	8.36
		48.55	35.1	8.36	29.5
4	0.071	28.52	23.6	11.1	9.35
		44.75	11.1	16.1	3.18
		45.27	9.35	3.18	6.31
5	0.0857	35.22	156	9.17	118
		24.94	9.17	31.1	-2.73
		57.72	118	-2.73	134
6	0.142	42.58	25.1	4.22	16.2
		49.55	4.22	12.9	-2.21
		52.88	16.2	-2.21	14.6
7	0.123	49.89	70.5	-29.3	50.2
		40.61	-29.3	46	-28.2
		59.53	50.2	-28.2	49.6
8	0.0686	62.34	20.8	-13.8	23.2
		41.47	-13.8	17.8	-17.2
		71.9	23.2	-17.2	31.5
9	0.0345	58.17	73.8	-35.5	85.7
		57.06	-35.5	58.6	-69.2
		62.88	85.7	-69.2	124
10	0.0878	52.41	26.5	-8.62	21.9
		48.86	-8.62	8.09	-8.59
		61	21.9	-8.59	20.6
11	0.0401	57.42	83.2	18.8	35
		36.42	18.8	52	-0.283
		106.9	35	-0.283	175
12	0.0491	75.74	24	-21.9	26.1
		32.33	-21.9	94	-20.5
		87.49	26.1	-20.5	39.9
13	0.0408	71.72	115	-67.9	183
		18.58	-67.9	79	-78.3
		91.09	183	-78.3	648
**(j)**					
**Cluster 10**					
**No**	**πk**	**μ**	**Σ**		
			L^*^	C^*^_ab_	***h***_**ab**_
1	0.047	20.1	47.2	19.4	41
		30.51	19.4	56.6	7.06
		42.36	41	7.06	47.3
2	0.132	21.12	53	7.18	58.5
		23.51	7.18	16.9	12.7
		44.32	58.5	12.7	87.4
3	0.12	15.66	8.58	6.84	5.27
		20.78	6.84	10.6	4.67
		43.55	5.27	4.67	16.6
4	0.119	31.75	114	23.8	63.6
		23.03	23.8	24.1	-6.76
		59.84	63.6	-6.76	108
5	0.07	21.12	12.4	5.81	2.31
		22.79	5.81	8.45	1.5
		66.73	2.31	1.5	5.49
6	0.0527	13.61	11.3	12	9.66
		17.91	12	16.5	6.15
		63.82	9.66	6.15	23.1
7	0.0448	55.24	303	0.527	310
		20.55	0.527	30.1	23.6
		73.48	310	23.6	478
8	0.0497	33.22	32.8	-1.92	2.8
		26.77	-1.92	2.47	1.92
		65.77	2.8	1.92	4.35
9	0.0881	45	95.7	-9.57	18.3
		29.24	-9.57	10.7	-1.27
		69.26	18.3	-1.27	9.03
10	0.0531	76.55	68.2	-52.2	21.2
		23.61	-52.2	95.8	0.0701
		80.24	21.2	0.0701	22.9
11	0.0608	91.78	13.6	-8.44	8.25
		6.639	-8.44	6.22	-2.28
		83.75	8.25	-2.28	50.3
12	0.0543	89.42	1.68	-1.82	2.57
		14.64	-1.82	8.76	-7.35
		112.4	2.57	-7.35	19.8
13	0.0331	90.29	18.7	-3.67	32.3
		19.47	-3.67	29.1	-33.9
		119.5	32.3	-33.9	123
**(k)**					
**Cluster 11**					
**No**	**πk**	**μ**	**Σ**		
			L^*^	C^*^_ab_	***h***_**ab**_
1	0.0873	24.82	21.9	11.2	11.4
		32.27	11.2	10.2	5.43
		46.75	11.4	5.43	10.1
2	0.0739	13.12	19.7	18.9	12.1
		17.94	18.9	23.8	11
		39.2	12.1	11	30.7
3	0.0433	9.028	14.3	16.9	25.2
		18.27	16.9	48.2	21.8
		31.63	25.2	21.8	57.2
4	0.143	35.85	54	18.2	15.1
		22.87	18.2	13.8	6.32
		81.98	15.1	6.32	11.4
5	0.077	41.96	220	65.6	171
		21.73	65.6	51.4	21.5
		73.64	171	21.5	394
6	0.12	33.07	76.3	9.2	51.4
		34.2	9.2	25.6	4.78
		50.2	51.4	4.78	46.3
7	0.0528	59.26	141	-117	228
		35.93	-117	235	-284
		75.21	228	-284	474
8	0.0411	84.29	15.4	2.27	-19.1
		8.911	2.27	11.5	-17.7
		118	-19.1	-17.7	116
9	0.107	73.96	74.8	-13.8	129
		18.42	-13.8	34.9	-44.8
		104.7	129	-44.8	366
10	0.0354	80.23	4.43	-3.19	4.49
		13.3	-3.19	5.79	-8.17
		130.2	4.49	-8.17	22.4
11	0.0741	82.09	15.1	-6.3	43.6
		7.432	-6.3	6.32	-21.6
		145	43.6	-21.6	363
12	0.0539	82.15	5.9	-1.65	15
		4.539	-1.65	1.73	-6.75
		181.2	15	-6.75	599
**(l)**					
**Cluster 12**					
**No**	**πk**	**μ**	**Σ**		
			L^*^	C^*^_ab_	***h***_**ab**_
1	0.0784	11.86	4.83	7.85	4.83
		22.29	7.85	14.6	7.25
		37.91	4.83	7.25	9.49
2	0.144	10.84	8.68	7.23	13.4
		13.65	7.23	7.1	9.49
		51.07	13.4	9.49	31.1
3	0.0539	18.8	9.04	7.7	5.53
		31.3	7.7	10.6	3.97
		43.63	5.53	3.97	6.34
4	0.0549	8.353	21.4	15	67
		8.408	15	13	51.7
		39.11	67	51.7	295
5	0.0352	21.3	51.3	21	66.3
		13.59	21	22.1	58.9
		54.57	66.3	58.9	312
6	0.097	7.691	15.3	20.7	29.1
		14.08	20.7	34.1	35.8
		33.24	29.1	35.8	73.9
7	0.0396	12.39	20.9	14.5	36.9
		24.24	14.5	39.6	14.7
		36.21	36.9	14.7	74.3
8	0.0738	5.496	1.54	1.9	-0.597
		7.849	1.9	4.16	-5.27
		37.58	-0.597	-5.27	64.4
9	0.105	18.67	15.7	7.14	7.59
		18.51	7.14	6.14	2.06
		57.71	7.59	2.06	17.1
10	0.0466	22.73	33.3	16.6	22.3
		32.11	16.6	38	7.73
		44.1	22.3	7.73	21.2
11	0.0449	49.15	284	111	106
		30.25	111	74.8	7.66
		84.45	106	7.66	123
12	0.0602	83.58	35.9	4.9	17.3
		40.03	4.9	29.9	0.171
		99.04	17.3	0.171	21.7
13	0.0372	91.48	3.71	-4.82	2.54
		37.56	-4.82	15.3	-5.63
		95.81	2.54	-5.63	3.45
14	0.0492	82.21	1.95	-5.1	0.819
		38.05	-5.1	23.8	-3.26
		100.5	0.819	-3.26	2.08
**(m)**					
**Cluster 13**					
**No**	**πk**	**μ**	**Σ**		
			L^*^	C^*^_ab_	***h***_**ab**_
1	0.0991	7.73	5.09	2.95	-12
		6.827	2.95	5.58	-1.97
		82.95	-12	-1.97	278
2	0.082	4.357	3.48	1.9	-14.1
		3.694	1.9	2.92	-18
		82.43	-14.1	-18	720
3	0.0422	26.05	71	8.21	11.8
		12.27	8.21	8.33	5.3
		104.1	11.8	5.3	7.84
4	0.0449	15.55	24.9	6.46	-5.92
		7.489	6.46	9.73	-9.99
		93.23	-5.92	-9.99	271
5	0.0381	12.34	11.9	10	1.51
		12.17	10	18	6.78
		94.43	1.51	6.78	105
6	0.0536	28.32	63.1	13.8	11.2
		18.47	13.8	34.9	15.2
		97.62	11.2	15.2	34.4
7	0.074	55.83	179	37.3	15.5
		26.39	37.3	34.1	2.62
		105	15.5	2.62	20.7
8	0.127	43.15	240	131	-14.8
		25.35	131	107	-21.8
		107.5	-14.8	-21.8	16.9
9	0.0934	67.43	82.2	18.3	-9.06
		35.91	18.3	20.6	-3.28
		107.2	-9.06	-3.28	7.68
10	0.135	78.75	14.4	3.91	0.469
		44.45	3.91	31.8	-10
		105.9	0.469	-10	9.01
11	0.106	78.94	0.723	0.948	-0.346
		41.65	0.948	8.23	-4.45
		107.5	-0.346	-4.45	6.22
**(n)**					
**Cluster 14**					
**No**	**πk**	**μ**	**Σ**		
			L^*^	C^*^_ab_	***h***_**ab**_
1	0.0764	28.44	95.5	29.4	118
		18.57	29.4	32.3	37.5
		56.51	118	37.5	220
2	0.056	24.15	35.4	2.84	20.1
		16.14	2.84	6.89	1.54
		59.25	20.1	1.54	58
3	0.0852	10.06	17.7	10.9	38.5
		10.77	10.9	14.9	13.1
		44.22	38.5	13.1	202
4	0.0498	13.53	22.1	14.9	38.8
		19.71	14.9	30.7	10.8
		43.31	38.8	10.8	108
5	0.038	4.167	6.76	3.88	40.1
		3.504	3.88	4.01	20.6
		39.26	40.1	20.6	515
6	0.0429	25.04	33.4	13.2	11.4
		22.69	13.2	12.9	1.42
		61.55	11.4	1.42	9.19
7	0.05	27.97	56.6	17.9	39.1
		33.36	17.9	21.5	9.99
		48.04	39.1	9.99	36.7
8	0.0339	37.47	270	37	22.9
		12.04	37	23.4	-3.66
		75.56	22.9	-3.66	398
9	0.0902	46.73	97.2	-0.232	55.4
		27.09	-0.232	24.1	0.492
		71.25	55.4	0.492	41.1
10	0.07	62.89	167	-33.3	171
		23.27	-33.3	71.7	-101
		84.11	171	-101	307
11	0.038	61.64	90.7	67.4	70.1
		52.81	67.4	169	-38.7
		76.62	70.1	-38.7	145
12	0.0639	75.67	55	-13.3	54.1
		16.72	-13.3	14.3	-7.93
		88.18	54.1	-7.93	67.6
13	0.0868	70.42	84.7	-3.68	42.1
		31.89	-3.68	95.5	-12.2
		89.69	42.1	-12.2	47.8
14	0.0705	88.54	18.3	1.23	7.04
		16.82	1.23	12.6	-9.54
		102.2	7.04	-9.54	17.5
15	0.0913	79.83	14.7	-4.71	2.6
		26.17	-4.71	36.7	-8.63
		97.63	2.6	-8.63	11.3
**(o)**					
**Cluster 15**					
**No**	**πk**	**μ**	**Σ**		
			L^*^	C^*^_ab_	***h***_**ab**_
1	0.0693	25.34	25.6	4.64	16.5
		29.74	4.64	18	2.47
		46.84	16.5	2.47	17.5
2	0.0509	11.26	20.9	13.1	34.8
		17.41	13.1	22.7	15.1
		38.8	34.8	15.1	91.8
3	0.0626	13.77	26.4	11.1	37.9
		11.48	11.1	11.9	9.26
		62.72	37.9	9.26	101
4	0.0857	50.54	199	-25.8	186
		31.37	-25.8	42.7	-33.5
		71.98	186	-33.5	202
5	0.0878	33.81	129	-10.9	151
		20.35	-10.9	34.2	-58.8
		76.56	151	-58.8	320
6	0.0511	65.85	195	36.2	76.5
		33.73	36.2	185	-89.8
		93.17	76.5	-89.8	124
7	0.121	90.91	0.539	-1.52	0.941
		27.61	-1.52	13.3	-4.09
		103.3	0.941	-4.09	2.23
8	0.161	78.57	17.2	2.58	8.52
		32.6	2.58	12.9	-2.8
		102.8	8.52	-2.8	9.8
9	0.0754	91.11	13.5	-3.31	10.2
		26.44	-3.31	13.8	-6.54
		102.2	10.2	-6.54	11.2
10	0.109	72.61	114	-4.41	48.9
		24.66	-4.41	37.6	-25.1
		95.15	48.9	-25.1	50.1
11	0.0516	84.62	1.29	-3.08	0.735
		30.69	-3.08	25.1	-8.3
		104.7	0.735	-8.3	4.6
**(p)**					
**Cluster 16**					
**No**	**πk**	**μ**	**Σ**		
			L^*^	C^*^_ab_	***h***_**ab**_
1	0.053	3.178	7.27	7.37	27.1
		4.671	7.37	9.14	33.2
		25.77	27.1	33.2	189
2	0.109	7.457	7.66	5.7	15.3
		7.645	5.7	8.53	-0.0991
		57.94	15.3	-0.0991	97.3
3	0.0845	14.49	24	11.1	31.5
		11.24	11.1	21.7	-4.76
		71.95	31.5	-4.76	229
4	0.0822	32.06	100	38.1	49.4
		19.95	38.1	41.5	-6.23
		87.39	49.4	-6.23	146
5	0.0673	4.425	2.67	1.2	3.86
		3.062	1.2	1.51	1.6
		65.56	3.86	1.6	315
6	0.0857	60.11	136	23.5	34.9
		33.04	23.5	38.3	-13.8
		93.39	34.9	-13.8	49.1
7	0.0438	80.85	47	-17.8	32.7
		32.96	-17.8	18.8	-10.3
		95.38	32.7	-10.3	26.5
8	0.0868	88.01	17.1	1.7	10.1
		42.11	1.7	12.2	-2.51
		98.38	10.1	-2.51	8.93
9	0.123	78.37	14.6	-13.3	1.94
		38.84	-13.3	20.1	-0.412
		94.68	1.94	-0.412	1.34
10	0.12	75.12	67.6	-23.5	28.7
		40.72	-23.5	31.7	-14.9
		95.07	28.7	-14.9	17.7
11	0.106	88.91	0.823	-0.703	1.12
		43.46	-0.703	6.03	-1.75
		99.44	1.12	-1.75	1.84
**(q)**					
**Cluster 17**					
**No**	**πk**	**μ**	**Σ**		
			L^*^	C^*^_ab_	***h***_**ab**_
1	0.0934	1.757	1.55	1.63	24
		2.504	1.63	2.17	26.9
		39.87	24	26.9	534
2	0.17	6.815	7.92	5.27	19.3
		7.339	5.27	8.8	-2.91
		56.23	19.3	-2.91	249
3	0.0911	3.277	2.14	1.35	-13.9
		2.305	1.35	1.86	-14
		74.9	-13.9	-14	1240
4	0.192	16.35	27.2	12.3	16.8
		14.75	12.3	19.1	2.33
		63.17	16.8	2.33	65.3
5	0.11	29.12	123	7.59	124
		16.86	7.59	46.5	13.2
		63.83	124	13.2	214
6	0.0386	0.8006	0.171	0.136	-3.13
		0.7935	0.136	0.211	-4.46
		95.03	-3.13	-4.46	420
7	0.127	68.41	317	-32.1	170
		16.53	-32.1	178	-14.2
		85.81	170	-14.2	435
8	0.0636	76.15	186	169	62.1
		71.75	169	170	47.8
		92.85	62.1	47.8	39.3
9	0.095	89.35	11.3	10	13.1
		89.18	10	8.91	11.6
		94.33	13.1	11.6	15.3
**(r)**					
**Cluster 18**					
**No**	**πk**	**μ**	**Σ**		
			L^*^	C^*^_ab_	***h***_**ab**_
1	0.0476	8.379	28.6	7.43	116
		3.279	7.43	2.57	38.9
		53.48	116	38.9	652
2	0.0563	5.994	8.89	3.81	3.2
		4.2	3.81	2.91	9.28
		76.43	3.2	9.28	210
3	0.0805	25.28	24.9	3.03	15.5
		11.2	3.03	1.87	5.72
		88.06	15.5	5.72	19.6
4	0.112	16.58	12.3	2.39	8.2
		7.358	2.39	2.13	5.32
		91.79	8.2	5.32	13.8
5	0.237	21.2	59.7	12.3	9.17
		10.17	12.3	7.94	6.08
		90.69	9.17	6.08	63.5
6	0.129	53.85	289	27.5	39.3
		16.14	27.5	22.7	-1.27
		100.9	39.3	-1.27	26.8
7	0.0621	85	43	-4	-2.88
		14.15	-4	13.9	2.55
		104.4	-2.88	2.55	6.82
8	0.0781	92.75	21.6	-7.8	3.04
		7.727	-7.8	9.51	-8.75
		111.5	3.04	-8.75	64.8
9	0.0409	92.8	20.7	-34.6	8.23
		22.8	-34.6	127	-19.5
		106.2	8.23	-19.5	6.6
10	0.046	83.62	29.3	20.2	3.34
		76.68	20.2	23.1	1.77
		101.2	3.34	1.77	0.948
**(s)**					
**Cluster 19**					
**No**	**πk**	**μ**	**Σ**		
			L^*^	C^*^_ab_	***h***_**ab**_
1	0.0678	9.571	4.95	3.47	10.8
		13	3.47	3.02	7.6
		56.61	10.8	7.6	33.3
2	0.0336	5.607	4.39	6.98	3
		9.399	6.98	12.4	2.21
		50.41	3	2.21	28.3
3	0.0417	4.763	2.13	2.2	1.73
		7.892	2.2	2.65	1.52
		41.03	1.73	1.52	12.6
4	0.0563	24.95	63.5	9.87	54.7
		21.78	9.87	36.4	7.91
		64.7	54.7	7.91	84
5	0.0828	27.61	44.4	25.9	11.7
		27.75	25.9	54	-4.38
		66.62	11.7	-4.38	12.3
6	0.0831	15.54	12.4	8.91	11
		18.03	8.91	14.6	7.12
		63.79	11	7.12	24.7
7	0.151	42.11	92.6	0.843	19
		28.62	0.843	10.1	1.35
		68.18	19	1.35	10.6
8	0.0752	57.23	232	52.3	86.9
		30.69	52.3	108	48.3
		78.23	86.9	48.3	57.9
9	0.0876	74.49	106	-38.6	54.6
		18.06	-38.6	27.9	-13
		78.51	54.6	-13	49.9
10	0.116	87.69	13.5	-8.21	12.6
		9.575	-8.21	7.66	-6.12
		84.86	12.6	-6.12	36.4
11	0.0465	89.96	9.83	-4.18	2.37
		6.625	-4.18	3.23	3.37
		81.79	2.37	3.37	67.7
**(t)**					
**Cluster 20**					
**No**	**πk**	**μ**	**Σ**		
			L^*^	C^*^_ab_	***h***_**ab**_
1	0.0577	5.365	8.67	7.38	0.361
		5.519	7.38	7.06	-1.76
		59.86	0.361	-1.76	90.1
2	0.0699	1.604	1.08	1.24	16.7
		2.551	1.24	1.78	20.8
		37.36	16.7	20.8	379
3	0.13	11.3	9.06	7.84	14.4
		14.86	7.84	9.95	7.9
		59.86	14.4	7.9	39.5
4	0.0839	5.061	4.51	5.15	14.1
		8.171	5.15	7.41	13.7
		44.92	14.1	13.7	91.2
5	0.17	22.45	19.5	0.64	1.34
		21.05	0.64	3.38	0.224
		65.41	1.34	0.224	6.64
6	0.0887	31.75	183	30.4	116
		20.07	30.4	66.5	25.6
		64.78	116	25.6	152
7	0.0583	28.34	25.7	-1.99	0.888
		26.22	-1.99	1.59	0.156
		64.36	0.888	0.156	3.14
8	0.107	16.51	11.7	3.21	5.4
		15.19	3.21	5.34	4.93
		65.4	5.4	4.93	18.3
9	0.0382	86.31	93.8	-25.5	109
		11.43	-25.5	28.7	-42.4
		89.53	109	-42.4	166
10	0.0641	91.36	11.9	-5.07	6.66
		8.602	-5.07	7.28	-0.354
		87.18	6.66	-0.354	38.1
11	0.0595	75.15	123	20.2	41
		40.72	20.2	248	21.6
		86.14	41	21.6	27.7
12	0.0347	86.22	8.15	12.7	8.35
		73.56	12.7	177	21.5
		90.73	8.35	21.5	9.86
**(u)**					
**Cluster 21**					
**No**	**πk**	**μ**	**Σ**		
			L^*^	C^*^_ab_	***h***_**ab**_
1	0.0479	4.719	3.86	2.48	-8.33
		3.69	2.48	2.96	-13.4
		52.05	-8.33	-13.4	543
2	0.0632	20.87	41.3	5.49	35
		17.1	5.49	7.78	4.83
		47.09	35	4.83	56.2
3	0.167	8.876	10.8	7.39	23.7
		9.31	7.39	8.78	14.4
		52.45	23.7	14.4	183
4	0.0356	11.91	31.3	30.6	40
		18.4	30.6	70.2	35.1
		32.95	40	35.1	71.5
5	0.103	16.83	9.66	0.943	2.21
		13.62	0.943	1.29	0.154
		67.46	2.21	0.154	11.3
6	0.0668	16.44	21	6.46	-5.75
		10.55	6.46	5.81	-2.79
		64.16	-5.75	-2.79	75.7
7	0.045	8.297	5.61	2.49	7.3
		7.55	2.49	1.25	3.19
		65.58	7.3	3.19	13.5
8	0.0903	28.25	71.5	-4.45	17.1
		15.8	-4.45	8.5	1.13
		63.87	17.1	1.13	53.6
9	0.114	4.179	1.68	1.1	2.12
		4.544	1.1	1.02	0.0985
		60.01	2.12	0.0985	65.9
10	0.0785	45.65	108	-18.6	45.4
		18.06	-18.6	11.9	-7.16
		71.81	45.4	-7.16	43.1
11	0.0375	84.22	50.2	-14.7	28.3
		11.98	-14.7	11.7	-5.32
		85.52	28.3	-5.32	35.3
**(v)**					
**Cluster 22**					
**No**	**πk**	**μ**	**Σ**		
			L^*^	C^*^_ab_	***h***_**ab**_
1	0.082	6.135	7.06	7.87	17.2
		8.317	7.87	11.9	17.6
		33.76	17.2	17.6	162
2	0.0986	28.67	50.5	15.3	41.8
		24.68	15.3	14.6	13.6
		48.87	41.8	13.6	53.2
3	0.1	25.62	34.2	7.25	21.3
		29.4	7.25	10.8	3.13
		46.91	21.3	3.13	20.1
4	0.146	17.14	15.1	11.5	11.1
		22.9	11.5	24.6	6.36
		42.75	11.1	6.36	23.1
5	0.0777	20.68	71.4	27.8	52.2
		15.43	27.8	30.5	16.7
		52.27	52.2	16.7	343
6	0.11	9.851	9.05	9.32	16.7
		15.5	9.32	14.7	14.7
		35.92	16.7	14.7	52.8
7	0.0369	3.537	0.824	0.813	-1.14
		3.379	0.813	1.84	-2.9
		38.49	-1.14	-2.9	399
8	0.044	26.42	28.8	14.9	2.1
		26.23	14.9	12.8	1.38
		69.02	2.1	1.38	6.86
9	0.0714	40.72	95.5	-28.7	57.6
		29.5	-28.7	28.1	-21
		55.9	57.6	-21	43.8
10	0.0634	53.6	147	-19.1	156
		19.33	-19.1	30.5	4.38
		71.02	156	4.38	271
11	0.0557	33.74	66.1	8.6	66.2
		24.14	8.6	8.36	7.36
		63.53	66.2	7.36	143
**(w)**					
**Cluster 23**					
**No**	**πk**	**μ**	**Σ**		
			L^*^	C^*^_ab_	***h***_**ab**_
1	0.124	24.48	34.5	6.58	28.6
		29.86	6.58	14.8	3.14
		47.91	28.6	3.14	37.2
2	0.0519	9.605	9.31	12.8	16.4
		16.75	12.8	21.1	20.3
		36.36	16.4	20.3	44.6
3	0.0785	16.68	24.9	12.5	39.3
		23.5	12.5	17.1	15.2
		45.13	39.3	15.2	80.5
4	0.0525	25.86	39.3	-4.02	13.3
		19.87	-4.02	1.71	-6.12
		72.05	13.3	-6.12	35.5
5	0.0504	26.73	129	83.2	134
		19.39	83.2	79.5	40.4
		68.75	134	40.4	757
6	0.14	18.31	21.7	11	14.5
		17.77	11	13.6	0.785
		68.95	14.5	0.785	47.2
7	0.0333	6.217	5.88	5.04	-1.83
		6.745	5.04	7.89	-26
		67.82	-1.83	-26	343
8	0.115	28.15	24.3	10.6	2.73
		24.38	10.6	24.3	-22.7
		66.68	2.73	-22.7	45
9	0.0529	37.04	57.7	-3.34	25.9
		23.83	-3.34	2.57	-4.92
		71.96	25.9	-4.92	28.3
10	0.0521	39.71	33.7	-5.39	16.4
		29.02	-5.39	2.48	-6.18
		70.25	16.4	-6.18	25.8
11	0.0652	36.34	187	-8.44	115
		10.54	-8.44	17	-54.6
		91.31	115	-54.6	372
12	0.0523	53.07	421	29	35.9
		6.988	29	8.89	43.6
		191.6	35.9	43.6	832
**(x)**					
**Cluster 24**					
**No**	**πk**	**μ**	**Σ**		
			L^*^	C^*^_ab_	***h***_**ab**_
1	0.0365	1.449	1.06	1.16	16.2
		2.449	1.16	1.84	18.2
		33.44	16.2	18.2	364
2	0.0953	5.192	4.44	4.36	12
		7.614	4.36	7.06	5.14
		46.65	12	5.14	120
3	0.142	10.26	7.49	6.44	14.6
		14.17	6.44	9.15	7.82
		57.52	14.6	7.82	47.5
4	0.0674	19.87	42.5	17.1	27.9
		15.11	17.1	15.5	11.1
		60.32	27.9	11.1	46.6
5	0.111	23.58	22.9	-1.23	0.832
		21.08	-1.23	1.17	0.529
		64.34	0.832	0.529	4.41
6	0.0861	32.07	43.7	-0.461	0.503
		24.16	-0.461	2.42	0.362
		66.7	0.503	0.362	8.61
7	0.13	16.26	12	8.62	8.21
		18.15	8.62	15.6	1.89
		63.71	8.21	1.89	18.9
8	0.0328	33.98	287	178	131
		24.48	178	123	91.6
		87.18	131	91.6	108
9	0.0836	46.32	149	0.449	49.8
		21.83	0.449	25.3	8.93
		69.91	49.8	8.93	39.7
10	0.0399	44.79	134	112	13.8
		38.25	112	104	11.9
		102.8	13.8	11.9	28
11	0.0403	75.51	86.4	-14.7	37.3
		14.16	-14.7	15.9	5.77
		77.15	37.3	5.77	50.7
12	0.0316	84.28	25.1	-0.791	28.8
		9.931	-0.791	1.15	0.208
		79.17	28.8	0.208	58.9
13	0.035	93.64	9.53	-4.38	21.4
		6.116	-4.38	3.84	-10.6
		90.94	21.4	-10.6	104

μ and ∑ in each component were arranged vertically in order of L*, C*_ab_, and *h*_*ab*_. Note that as the components that have πk < 3% were excluded from analysis, the number of components in each cluster differs from those in Table 4. (a)-(x) show the clusters 1–24, respectively.

From an interpretation of Table 4 according to the definition of Table 2, the color combinations of the clusters tend to exhibit the following four patterns:

1. contrasting lightness, similar chroma, and similar hue
2. contrasting lightness, contrasting chroma, and similar hue
3. similar lightness, similar chroma, and complementary hue
4. similar lightness, similar chroma, and similar hue

**Fig 5:**
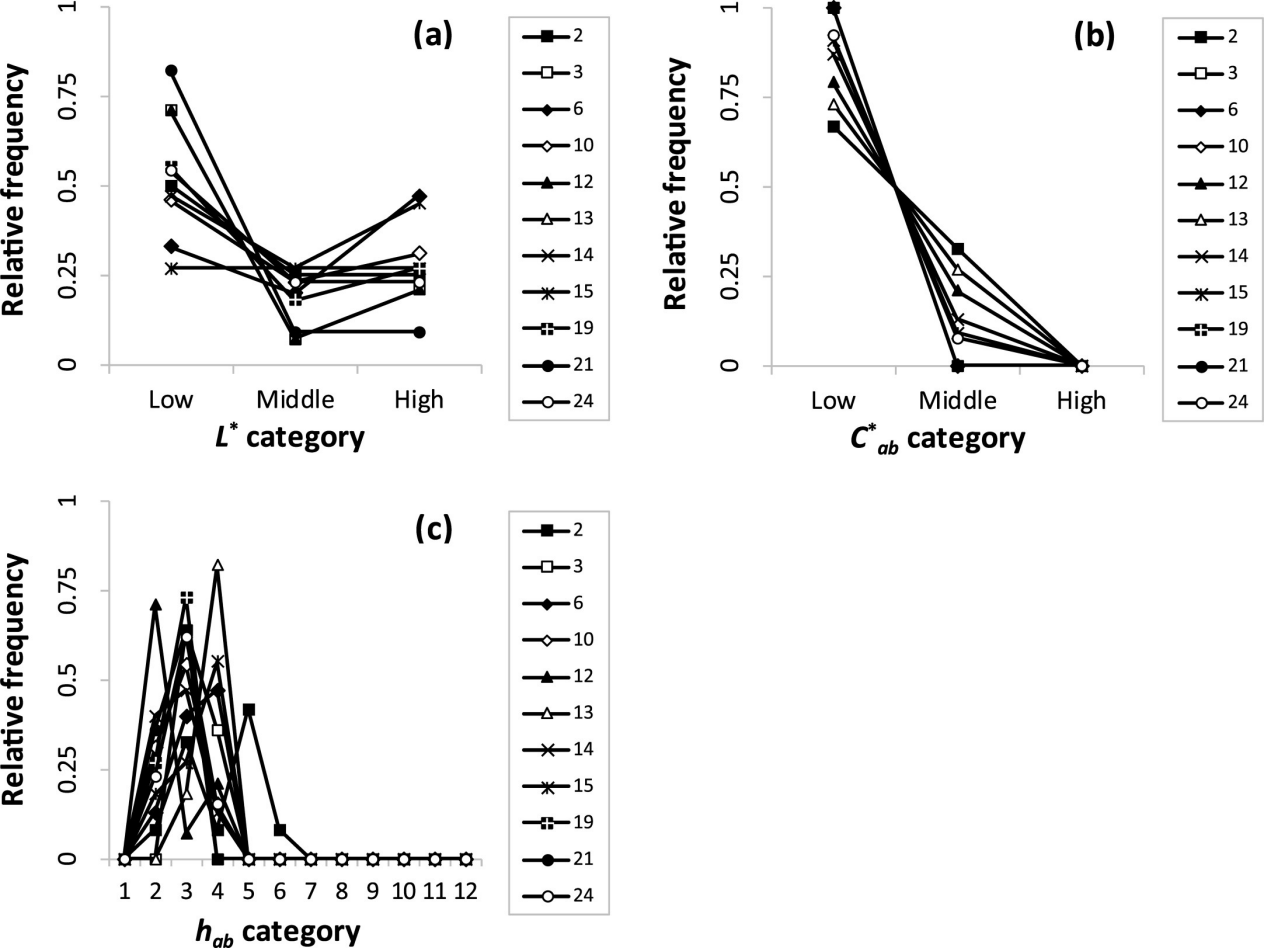
Relative frequencies of (a) L*, (b) C*_ab_, and (c) *h*_*ab*_ categories in color combination pattern 1.

**Fig 6:**
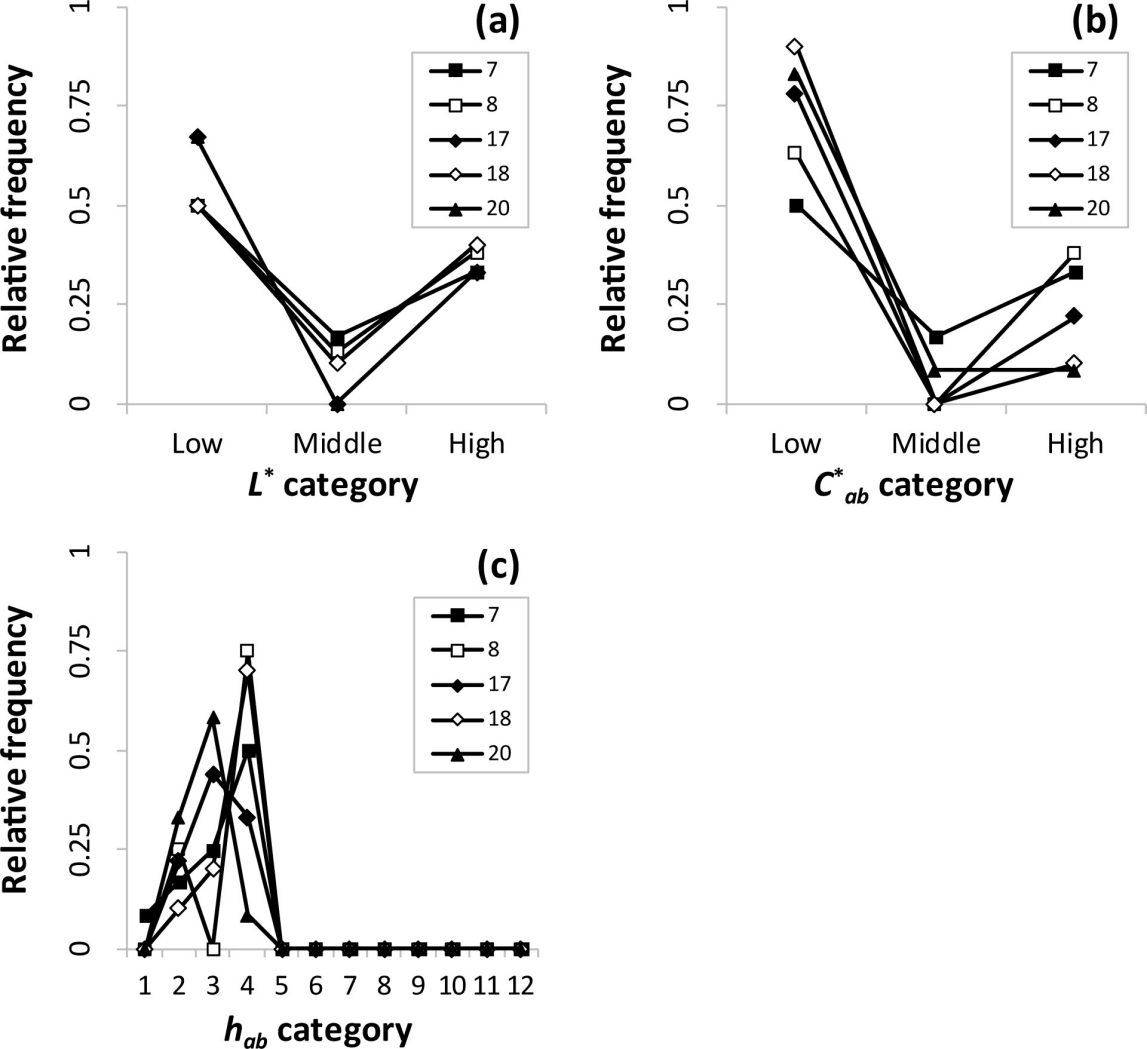
Relative frequencies of (a) L*, (b) C*_ab_, and (c) *h*_*ab*_ categories in color combination pattern 2.

**Fig 7:**
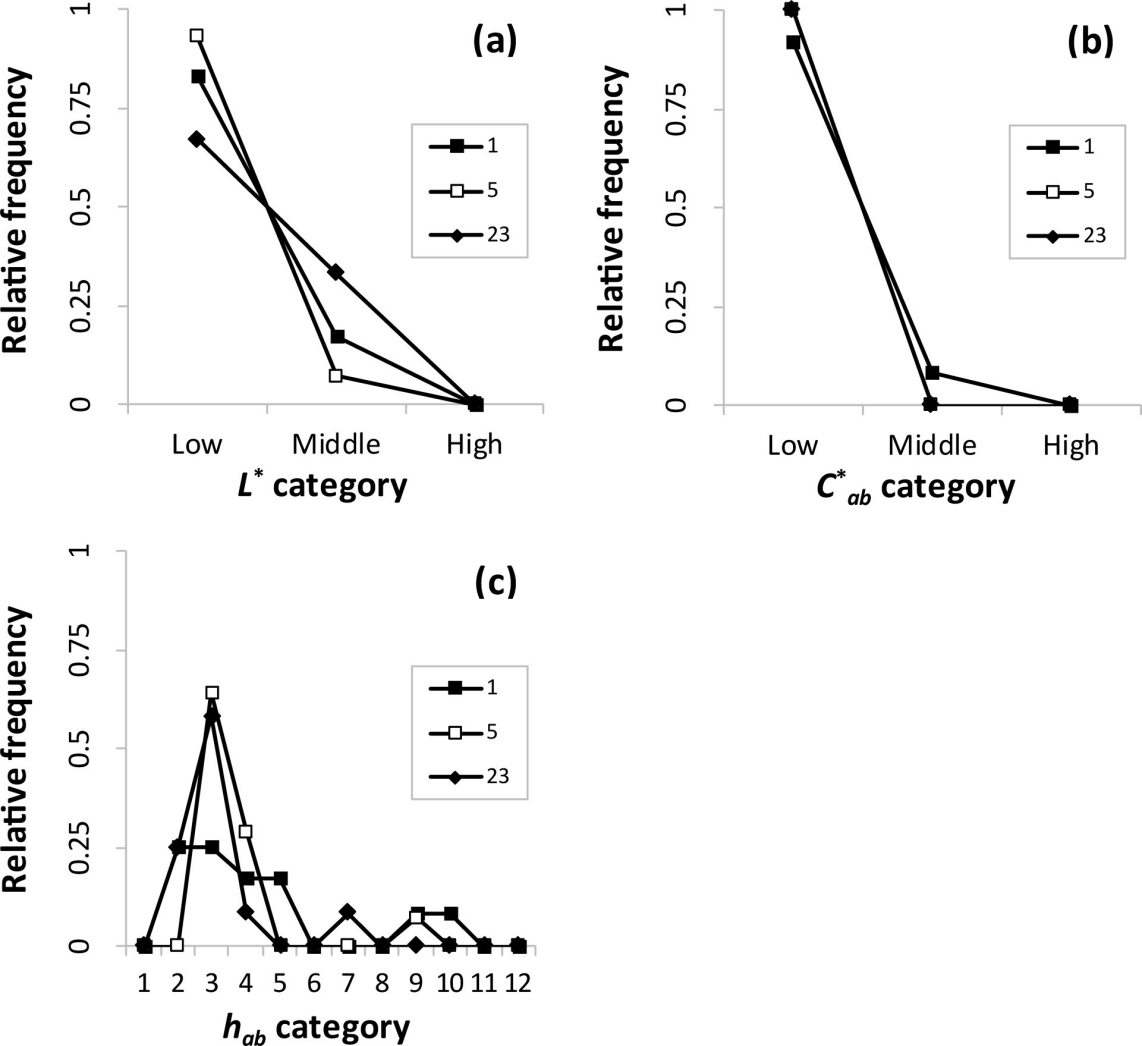
Relative frequencies of (a) L*, (b) C*_ab_, and (c) *h*_*ab*_ categories in color combination pattern 3.

Figs 5–7 depict the relative frequencies of CIELCh categories in each cluster of the color combination patterns 1–4. In Fig 5, 11 clusters were included in pattern 1. In terms of lightness (Fig 5(A)), low-category colors were more frequent than their high-category counterparts; there were a few mid-category colors. This result indicates the dominant low lightness and contrasting lightness combinations. In terms of chroma (Fig 5(B)), the low categories were the most frequent. There were a few middle categories, but no high categories. This indicates the presence of dominant low chroma and similar chroma combinations. In terms of hue (Fig 5(C)), the hues 2–4 occurred relatively more frequently; additionally, no further than the sixth hue was present. This indicates the dominant orange to yellow (the ranges of hue names in CIELCh were determined based on [28, 29]) and similar hue combinations. Therefore, these clusters mainly have contrasting lightness, similar chroma, and similar hue combinations.

As shown in Fig 6, five clusters were included in pattern 2. In terms of lightness (Fig 6(A)), the same tendency as that in Fig 5(A) was observed. As for chroma (Fig 6(B)), the same tendency as that of lightness appeared. These results indicate the dominant low lightness (chroma) and contrasting lightness (chroma) combinations. In terms of hue (Fig 6(C)), the hues 2–4 occurred relatively more frequently, and no further than the fourth hue appeared. This indicates the dominant orange to yellow and similar hue combinations. Therefore, these clusters mainly have contrasting lightness, contrasting chroma, and similar hue combinations.

As shown in Fig 7, three clusters were included in pattern 3. In terms of lightness (Fig 7(A)), the low categories were the most frequent. There were few middle categories, and no high categories. In terms of chroma (Fig 7(B)), the same tendency as that of lightness appeared. These indicate the dominant low lightness (chroma) and similar lightness (chroma) combinations. In terms of hue (Fig 7(C)), the hues 2–4 occurred relatively more frequently, and there were a few ninth and tenth hues. These indicate the dominant orange and complementary hue combinations. Thus, these clusters mainly have similar lightness, similar chroma, and complementary hue combinations.

**Fig 8:**
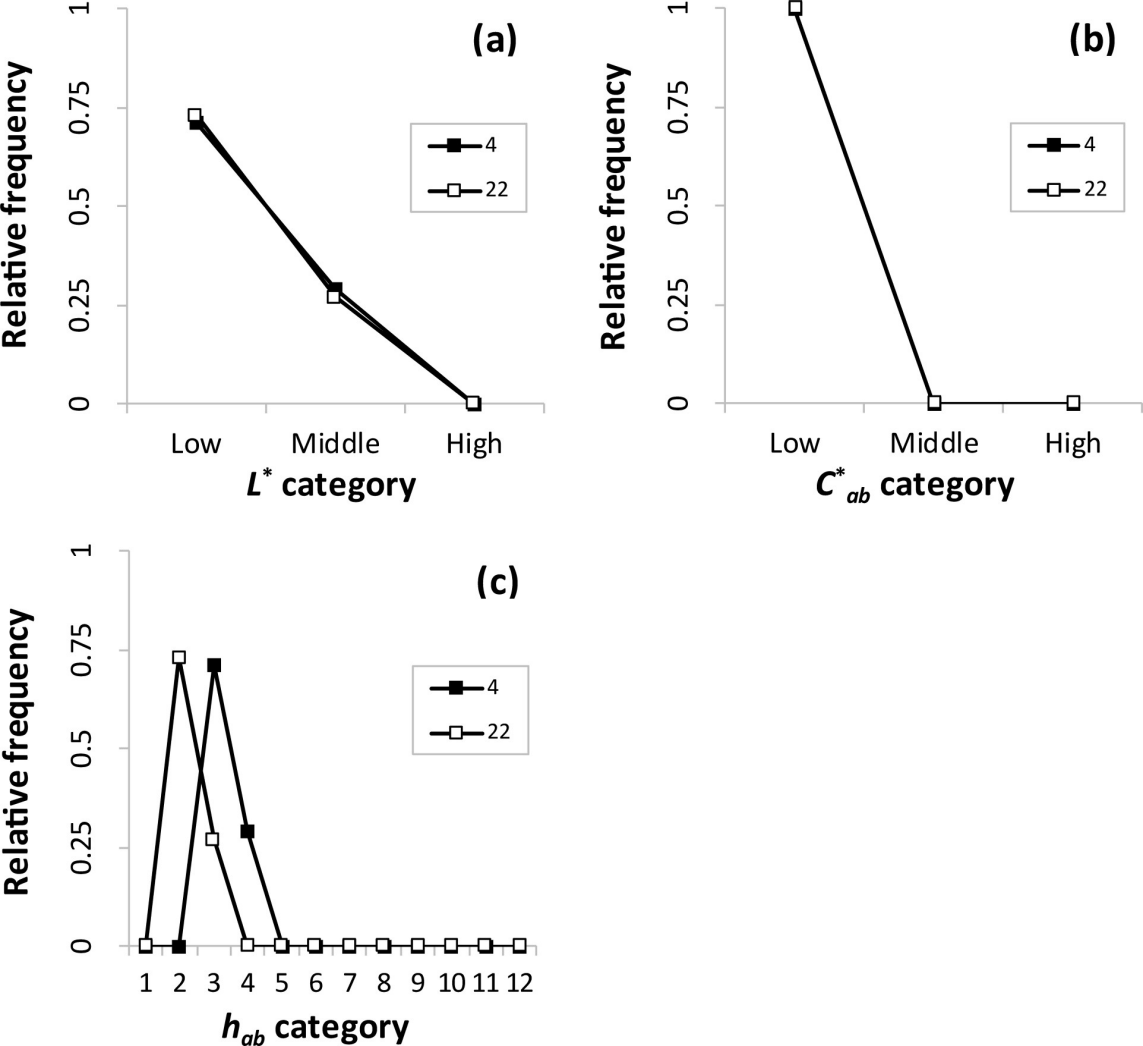
Relative frequencies of (a) L*, (b) C*_ab_, and (c) *h*_*ab*_ categories in color combination pattern 4.

In Fig 8, two clusters were included in pattern 4. In terms of lightness and chroma (Fig 8(A) and 8(B)), the same tendencies as those in Fig 7(A) and 7(B) appeared, respectively. These indicate the dominant low lightness (chroma) and similar lightness (chroma) combinations. In terms of hue (Fig 8(C)), the same tendency as that in Fig 6(C) appeared. These indicate the dominant orange and similar hue combinations. Accordingly, these clusters mainly have similar lightness, similar chroma, and similar hue combinations.

The above four patterns did not appear in clusters 9, 11, and 16. In cluster 9 (Table 4(i)), the middle lightness and chroma were dominant. The hues 2–4 were frequent. Thus, cluster 9 only has similar hue combinations. In cluster 11 (Table 4(k)), the middle and high lightness were frequent. The low chroma was most frequent. There were a few middle chroma and no high chroma. The hues 2–4 were frequent. Thus, cluster 11 mainly had similar chroma and similar hue combinations. In cluster 16 (Table 4(p)), the low and high lightness were frequent, along with the low and middle chroma. Further, the hues 2–4 were frequent. Therefore, cluster 16 mainly has contrasting lightness and similar hue combinations.

In addition, these color combination patterns are not always consistent with the characteristics of the dendrogram in Fig 4. In this dendrogram, the higher the similarity of colors of images, the greater the belongingness of the corresponding images to the adjacent branches. However, a few clusters that have the same color combination pattern are found farther from each other. Further, in Fig 4, the conspicuous chromatic colors of the clusters that belong to the same branch are similar. In fact, clusters 7 and 8, which belong to the same branch, consist of images of yellow and yellow-green butterflies. Similarly, clusters 1 and 2 consist of images of blue-green butterflies; clusters 12–20 consist of images of yellow butterflies. (Note that the relative areas of the conspicuous chromatic colors are different.) Therefore, the results of the image clustering in this study could be influenced by the conspicuous chromatic colors as well.

## Discussion and limitations

We obtained the holistic color combination rules of human-preferred *Papilionidae* butterflies for 4 different categories, as mentioned previously. In the previous psychological color harmony studies, the following robust color harmony principles were invariably obtained: “High lightness,” “Unequal lightness values” (large lightness difference), “Equal chroma” (same or similar in chroma color), and “Equal hue” (same or similar hue color) [1–4]. The above three principles, except “High lightness,” qualitatively agreed with the results of our previous work, i.e., contrasting lightness, similar chroma, and similar hue [6]. In addition to our previous work, the color combination pattern 1 is also consistent with the above three principles (e.g., “Unequal lightness values,” “Equal chroma,” and “Equal hue”) in this study. As they appeared most frequently in the clusters, the contrasting lightness, similar chroma, and similar hue are the most dominant color combinations of human-preferred *Papilionidae* butterflies.

Similar lightness, contrasting chroma, and complementary hue differ from the above principles; however, they appeared in the color combination patterns 2–4 and in the minority of results in our previous work [6]. The similar lightness and complementary hue qualitatively agree with the “equal lightness” and “complementary hue” that are part of the conventional color harmony principles [1]. The contrasting chroma appears in the results of Chuang and Ou as well, and their results exhibited large 95% confidence intervals [18]. It is likely that these color combinations did not appear because the color appearance attributes were handled independently and those results were simplified in previous psychological color harmony studies. In contrast, this study investigated the results by integrating color appearance attributes. Therefore, these color combinations may not be universal, but may harmonize to a limited extent. The conditions for this are as follows. The similar lightness harmonizes by combining similar chroma. The contrasting chroma harmonizes by combining contrasting lightness and similar hue. The complementary hue harmonizes by combining similar lightness and similar chroma.

Furthermore, this study has several limitations. Initially, the conditions that harmonize similar lightness, contrasting chroma, and complementary hue were shown. As these color combination rules were obtained from the human-preferred *Papilionidae* butterflies for which the color harmony was demonstrated in our previous work, these rules are valid. However, we cannot conclude these results for color harmony theories based on limited samples. Psychological experiments to investigate the harmonies of the color combinations under those conditions are required in future work.

Subsequently, the color combination types were defined based on the ranges of segmented color space. These definitions of similar and contrasting color combinations may not always agree with perception. To achieve a more perceptual color combination analysis, the threshold of similarity and contrast must be determined experimentally in future work.

Moreover, it is suggested that the results of image clustering based on the human visual perception in this study were influenced by the conspicuous chromatic colors. Therefore, in the future work, the conspicuous chromatic colors should also be included in the color combination analysis.

Similarly, the concepts of the methods of our previous study and this study must be regulated, to compare the results. In our previous study, we employed a simple and conventional approach to obtain standard results (i.e., data comparable with the results of the future works) because the color combination analysis method has not been established until now. On the other hand, because a fuzzy logic is applied to color image segmentation and color planning system based on the image [8, 30–32], the same can be applied to color combination analysis in future works as well.

Finally, the method used in this study can be improved to enable application to the color combination analysis of other aesthetic objects (e.g., flowers, jewelries, etc.). We will analyze and clarify the color combination rules of other aesthetic objects in our future works. If the aesthetic objects reflect the human psychological aesthetic responses, the color combination rules of other aesthetic objects may agree with the results of this study and previous color harmony studies. Moreover, the color combination rules peculiar to other aesthetic objects could also be shown.

## Conclusion

In this study, we aimed to clarify the perceptual holistic color combination rules of human-preferred *Papilionidae* butterflies. To achieve this, the *Papilionidae* butterfly images were classified via hierarchical density-based spatial clustering based on experimentally obtained perceptual color similarities. The color combinations of the clustered images were determined based on representative colors extracted by the GMM with minimum message length. We obtained the following holistic color combination rules of *Papilionidae*:

1. contrasting lightness, similar chroma, and similar hue
2. contrasting lightness, contrasting chroma, and similar hue
3. similar lightness, similar chroma, and complementary hue
4. similar lightness, similar chroma, and similar hue

The first rule agrees with the results of our previous work and some of the most robust harmony principles of psychological studies. The other rules suggest that similar lightness, contrasting chroma, and complementary hue harmonize to a limited extent. Future studies will focus on the following issues:

- Experimental verification of the harmonies of the above color combination rules, except the first rule
- Color combination analysis of other aesthetic objects
- Further perceptual analysis based on the conspicuous chromatic colors and a fuzzy logic
- Experimental clarification of the threshold for similar and contrasting color combinations
